# Association between early intensive care or coronary care unit admission and post-discharge performance of activities of daily living in patients with acute decompensated heart failure

**DOI:** 10.1371/journal.pone.0251505

**Published:** 2021-05-10

**Authors:** Masato Kanda, Kazuya Tateishi, Atsushi Nakagomi, Togo Iwahana, Sho Okada, Hiroyo Kuwabara, Yoshio Kobayashi, Takahiro Inoue

**Affiliations:** 1 Department of Cardiovascular Medicine, Chiba University Graduate School of Medicine, Chiba, Japan; 2 Takemi Program in International Health, Harvard T.H. Chan School of Public Health, Boston, Massachusetts, United States of America; 3 Department of Healthcare Management Research Center, Chiba University Hospital, Chiba, Japan; Erasmus Medical Centre: Erasmus MC, NETHERLANDS

## Abstract

The management of acute decompensated heart failure often requires intensive care. However, the effects of early intensive care unit/coronary care unit admission on activities of daily living (ADL) in acute decompensated heart failure patients have not been precisely evaluated. Thus, we retrospectively assessed the association between early intensive care unit admission and post-discharge ADL performance in these patients. Acute decompensated heart failure patients (New York Heart Association I–III) admitted on emergency between April 1, 2014, and December 31, 2018, were selected from the Diagnosis Procedure Combination database and divided into intensive care unit/coronary care unit (ICU) and general ward (GW) groups according to the hospitalization type on admission day 1. The propensity score was calculated to create matched cohorts where admission style (intensive care unit/coronary care unit admission) was independent of measured baseline confounding factors, including ADL at admission. The primary outcome was ADL performance level at discharge (post-ADL) defined according to the Barthel index. Secondary outcomes included length of stay and total hospitalization cost (expense). Overall, 12231 patients were eligible, and propensity score matching created 2985 pairs. After matching, post-ADL was significantly higher in the ICU group than in the GW group [mean (standard deviation), GW vs. ICU: 71.5 (35.3) vs. 78.2 (31.2) points, P<0.001; mean difference: 6.7 (95% confidence interval, 5.1–8.4) points]. After matching, length of stay was significantly shorter and expenses were significantly higher in the ICU group than in the GW group. Stratified analysis showed that the patients with low ADL at admission (Barthel index score <60) were the most benefited from early intensive care unit/coronary care unit admission. Thus, early intensive care unit/coronary care unit admission was associated with improved post-ADL in patients with emergency acute decompensated heart failure admission.

## Introduction

Heart failure is a common cause of mortality and morbidity [1, 2] and acute decompensated heart failure (ADHF) is one of the common reasons for emergency department (ED) visits [2]. The underlying heart diseases, comorbidities, causes of worsening conditions, and clinical pathology of ADHF are diverse [3]; therefore, the management of heart failure requires various treatment modalities, including intensive care which is limited in resources and puts a burden on the medical economy [4, 5].

In many cases of severe ADHF, the rapid initiation of intense invasive treatments, such as mechanical respiration, hemodialysis, intra-artery balloon pumping (IABP), or extracorporeal membrane oxygenation (ECMO), is required, and these treatments are best performed in intensive care unit (ICU) settings [3, 6]. The European Society of Cardiology 2016 guidelines recommended that ADHF patients with signs or symptoms of hypoperfusion, significant vital instability, or need for intubation should be considered for ICU or coronary care unit (CCU) admission [3, 7]. In some cases, a patient’s vital signs may be normal; however, several worsening stages could require urgent definitive intervention [6, 8], in which the ICU/CCU are probable places for admission. However, the inappropriate usage of the ICU/CCU might lead to an increase in medical costs [5]. The definite criterion for ICU/CCU admission is still a matter of conflict, and there are wide regional and international variations in the admission rates to ICU for ADHF patients[6, 9].

The aforementioned studies evaluated ADHF prognosis using short-term mortality, the occurrence of life-threatening events, and readmission as primary outcomes. However, ADHF has a substantial impact on activities of daily living (ADL), and ADL performance levels at discharge (post-ADL) can provide additional prognostic value for long-term mortality and readmission [10–12]. In addition, as ADL level is closely related to the quality of life [13, 14], the recovery of ADL is a significant goal for the social well-being of ADHF patients. Rapid recovery from ADHF via early intensive therapy might contribute to ADL recovery. However, unnecessary restrictions in the ICU/CCU might worsen physical activity. Therefore, in this study, we sought to assess the association between early ICU/CCU admission and post-discharge performance of ADL in patients with ADHF, using a large database in Japan.

## Materials and methods

### Data source

This study was approved by the ethics committee of Chiba University Hospital (number-3309). All procedures were in adherence to the 1975 Declaration of Helsinki. The requirement for informed consent was waived because of the anonymous nature of the data. The Diagnosis Procedure Combination (DPC) database contains administrative information obtained during acute phase hospitalization, and it is used for reimbursement in the Per-Diem Payment System. The database contains patient information on demographics, such as age, sex, height, weight, the most resource-consuming disease, in-hospital death, other major diagnoses and comorbidities, New York Heart Association (NYHA) class, consciousness level, and ADL status, as well as prescribed medications, treatment procedures, and other hospital-related information. This study used the DPC database, and data were regularly collected from the hospitals that voluntarily participated in the DPC system.

### Selection of study population

The detailed flow-chart of the study design is presented in S1 Fig. This retrospective study enrolled patients aged ≥20 years with heart failure and emergency admission by identifying cases in which the most resource-consuming disease during hospitalization was identified as heart failure (DPC code: 050130) based on the International Classification of Diseases (ICD-10) coding. The 61532 patients admitted to the 258 hospitals where an ICU was available between April 1, 2014, and December 31, 2018, were included. We excluded patients with missing body mass index or ADL data and those who died during admission. For the NYHA category, we mainly evaluated patients with NYHA I–III in order to exclude patients with severe ADHF.

### Outcomes

The primary outcome was post-ADL. The ADL status was evaluated using the Barthel Index (BI) [15–17]. The BI consists of 10 items concerning functional capability regarding ADL (e.g., feeding, dressing, and chair/bed transfer). The score ranges from 0 (totally dependent when carrying out ADL) to 100 (fully independent in carrying out ADL). For stratified analysis, we applied a BI score of 60 as a meaningful ADL deterioration threshold [17].

The secondary outcomes were the total length of stay (LOS: days from admission to discharge) and total hospitalization cost (expense) based on reimbursement of treatment costs from the DPC system.

### Exposure and baseline variables

The exposure variable was ICU/CCU use on hospital admission. Admission to the ICU/CCU was defined as the presence of an ICU/CCU revenue center code in the administrative DPC data. We defined general ward (GW) admission as the lack of any ICU/CCU revenue center code. Patients were divided into the ICU/CCU (hereafter referred to as ICU) and GW groups according to the type of hospitalization room on admission day 1.

We included the following baseline characteristics: age, sex, body mass index, ambulance use, weekend admission, history of heart failure admission, pre-ADL, NYHA classification on admission, impairment in consciousness (judging from the Japan Coma Scale score [18]) on admission, medical history (hypertension, diabetes mellitus, dyslipidemia, cerebrovascular disease, atrial fibrillation, cardiac arrhythmia [excluding atrial fibrillation], ischemic heart disease, valvular heart disease, dilated cardiomyopathy, peripheral vascular disease, pulmonary hypertension, pneumonia, asthma or chronic obstructive pulmonary disease, chronic renal disease, anemia, cancer, disuse syndrome, and dementia [judging from impairment in cognitive function]), and hospital volume. We calculated the average number of annual hospital ADHF admissions for each hospital, and the hospitals were divided into quartiles based on the number of admissions [19].

### Post-admission treatments

The following post-admission treatments were also recorded: medications such as inotrope, diuretic, aldosterone antagonist, angiotensin-converting enzyme inhibitor (ACE-I), angiotensin II receptor blocker, beta-blocker, class III antiarrhythmic, antiplatelet, anticoagulant, digitalis, vasodilator, calcium-channel blocker, and statin; hemodiafiltration; mechanical ventilation; cardiopulmonary resuscitation; cardioversion; IABP; percutaneous coronary intervention; ablation; coronary artery bypass grafting; temporary pacing; ECMO.

### Statistical analyses

All analyses were conducted using Python version 2.7.15 (Van Rossum G, Drake Jr FL. Python reference manual. Centrum voor Wiskunde en Informatica Amsterdam) and R version 3.6.2 (R Foundation for Statistical Computing, Vienna, Austria).

#### Propensity score analysis

We applied a propensity score approach because non-random assignment to either an ICU or a GW in this observational study was likely to produce a selection bias [20, 21]. The propensity score for each patient was the conditional probability of ICU admission on day 1, estimated using a logistic regression model with ICU admission as the dependent variable and all measured baseline variables as the independent variables. Each patient in the ICU group was matched with one patient in the GW group with the closest estimated propensity score within a caliper (≤0.20 of the pooled standard deviation [SD] of estimated logits), based on the nearest-neighbor method without replacement. For the comparison of baseline characteristics, absolute values >10% of standardized differences were considered as demonstrating significant imbalance.

The implement of each treatment after admission, post-ADL, LOS, and expense before and after matching were compared using the Pearson’s chi-squared test, Welch’s t-test, Student’s t-test, and Mann-Whitney U-test, as appropriate. The difference in means (and 95% confidence intervals [CIs]) of post-ADL between ICU and GW groups was calculated using Welch’s t-test. P values < 0.05 were considered statistically significant [20].

#### Sensitivity analyses

We conducted a series of sensitivity analyses. First, we performed propensity score matching analyses among patients with 1) NYHA I–II, 2) NYHA I–IV, 3) NYHA I–IV plus NYHA-unknown, 4) NYHA IV, and 5) NYHA I–IV, excluding patients with respirator treatment on admission day 1. Second, we conducted propensity score matching analyses where changes in ADL performance levels from admission to discharge were used as an outcome among patients with NYHA I–III. Third, multivariate linear regression analyses were performed using the entire (unmatched) patient cohort with NYHA I–III. In the multiple linear regression analysis, post-ADL was used as the dependent variable, and all baseline variables, including pre-ADL and ICU admission, were selected as independent variables. Fourth, we performed propensity score matching analysis in NYHA I–III patients including those who died in hospital.

#### Stratified analyses

We first performed a test for interaction with ICU admission using multivariable models to identify stratified groups with statistically significant differences [22, 23]. We then performed analyses of variables in stratified groups with a significant interaction. In each stratified group, we created propensity scores using the conditional probability of ICU admission on day 1, estimated by a logistic regression model with ICU admission as the dependent variable and all measured baseline variables, except for stratified group variable, as independent variables. After propensity score matching, the post-ADLs of patients in the ICU and GW were compared using Welch’s t-test, Student’s t-test, or Mann-Whitney U-test, as appropriate.

## Results

### Study patients

After applying the exclusion criteria, a total of 12231 patients were eligible (S1 Fig). Of these patients, 3523 (28.8%) were admitted to the ICU and 8708 (71.2%) were admitted to the GW on day 1. Propensity score matching created 2985 pairs.

### Baseline characteristics

The baseline characteristics of the study population are summarized in Table 1. In the pre-match cohort, the ICU group had more ambulance use, weekend admission, lower pre-ADL, higher NYHA class, more impairment of consciousness, and more admission to high-volume hospitals than the GW group. In the matched cohort, the baseline characteristics were balanced, including pre-ADL.

**Table 1:** Baseline characteristics of the pre-match and matched samples.

Variable	Before propensity score matching				After propensity score matching			
	GW (n = 8708)	ICU (n = 3523)	Absolute standard-ized difference, %	P-value	GW (n = 2985)	ICU (n = 2985)	Absolute standard-ized difference, %	P-value
**Age (years), mean (SD)**	77.7 (12.4)	77.2 (12.5)	3.7	0.063	77.8 (12.8)	77.6 (12.4)	1.1	0.536
**Male**	4816 (55.3%)	1912 (54.3%)	2.1	0.308	1593 (53.3%)	1605 (53.7%)	0.8	0.775
**BMI (kg/m**^**2**^**), mean (SD)**	22.8 (4.8)	22.6 (4.7)	3.3	0.101	22.6 (4.7)	22.6 (4.8)	1.6	0.536
**Ambulance use**	2130 (24.5%)	1963 (55.7%)	67.3	<0.001	1479 (49.5%)	1465 (49.0%)	0.9	0.717
**Weekend admission**	1324 (15.2%)	823 (23.4%)	20.8	<0.001	677 (22.7%)	643 (21.5%)	2.7	0.289
**Pre-history of HF admission**	2294 (26.3%)	865 (24.6%)	4.1	0.040	753 (25.2%)	746 (25.0%)	0.5	0.835
**Pre-ADL score, mean (SD)**	65.6 (37.0)	39.0 (39.4)	69.7	<0.001	44.7 (38.6)	44.5 (39.7)	0.5	0.837
**NYHA class at admission**			11.0	<0.001			2.7	0.574
**I**	995 (11.4%)	394 (11.2%)			350 (11.7%)	329 (11.0%)		
**II**	3404 (39.1%)	1202 (34.1%)			1015 (34.0%)	1045 (35.0%)		
**III**	4309 (49.5%)	1927 (54.7%)			1620 (54.3%)	1611 (54.0%)		
**Impairment in consciousness**	885 (10.2%)	688 (19.5%)	26.6	<0.001	519 (17.4%)	519 (17.4%)	<0.1	1.000
**Comorbidity**								
**Hypertension**	4463 (51.3%)	1893 (53.7%)	5.0	0.013	1567 (52.5%)	1601 (53.6%)	2.3	0.378
**Diabetes**	2475 (28.4%)	1041 (29.5%)	2.5	0.213	871 (29.2%)	882 (29.5%)	0.8	0.755
**Dyslipidemia**	1786 (20.5%)	859 (24.4%)	9.3	<0.001	702 (23.5%)	704 (23.6%)	0.2	0.951
**Cerebrovascular disease**	520 (6.0%)	245 (7.0%)	4.0	0.042	205 (6.9%)	205 (6.9%)	<0.1	1.000
**Atrial fibrillation**	2883 (33.1%)	1033 (29.3%)	8.2	<0.001	942 (31.6%)	892 (29.9%)	3.6	0.161
**Cardiac arrhythmia**	3272 (37.6%)	1197 (34.0%)	7.5	<0.001	1071 (35.9%)	1023 (34.3%)	3.4	0.193
**IHD**	2328 (26.7%)	1084 (30.8%)	8.9	<0.001	884 (29.6%)	892 (29.9%)	0.6	0.821
**VHD**	1535 (17.6%)	539 (15.3%)	6.3	0.002	442 (15.8%)	469 (15.7%)	0.3	0.915
**DCM**	404 (4.6%)	149 (4.2%)	2.0	0.323	123 (4.1%)	132 (4.4%)	1.5	0.565
**PVD**	167 (1.9%)	66 (1.9%)	0.3	0.871	56 (1.9%)	56 (1.9%)	<0.1	1.000
**PH**	119 (1.4%)	28 (0.8%)	5.5	0.009	28 (0.9%)	26 (0.9%)	0.7	0.785
**Congenital heart disease**	57 (0.7%)	18 (0.5%)	1.9	0.357	19 (0.6%)	17 (0.6%)	0.9	0.738
**Pneumonia**	399 (4.6%)	225 (6.4%)	7.9	<0.001	179 (6.0%)	182 (6.1%)	0.4	0.871
**COPD or asthma**	393 (4.5%)	173 (4.9%)	1.9	0.343	146 (4.9%)	135 (4.5%)	1.7	0.502
**CRD**	1261 (14.5%)	541 (15.4%)	2.5	0.216	430 (14.4%)	447 (15.0%)	1.6	0.534
**Anemia**	589 (6.8%)	219 (6.2%)	2.2	0.270	183 (6.1%)	189 (6.3%)	0.8	0.748
**Cancer**	545 (6.3%)	186 (5.3%)	4.2	0.039	167 (5.6%)	162 (5.4%)	0.7	0.777
**Disuse syndrome**	116 (1.3%)	34 (1.0%)	3.4	0.095	39 (1.3%)	32 (1.1%)	2.2	0.403
**Dementia**	2454 (28.2%)	921 (26.1%)	4.6	0.022	855 (28.6%)	829 (27.8%)	1.9	0.455
**Annual hospital volume, case/year**			53.4	<0.001			3.9	0.510
**Quartile 1 (<59)**	761 (8.7%)	33 (0.9%)			24 (0.8%)	33 (1.1%)		
**Quartile 2 (59–126)**	1654 (19.0%)	344 (9.8%)			360 (12.1%)	336 (11.3%)		
**Quartile 3 (127–210)**	2363 (27.1%)	832 (23.6%)			752 (25.2%)	761 (25.5%)		
**Quartile 4 (≥211)**	3930 (45.1%)	2314 (65.7%)			1849 (61.9%)	1855 (62.1%)		

Data are shown as number (%) unless otherwise stated.

BMI: body mass index; COPD: chronic obstructive pulmonary disease; CRD: chronic renal disease; DCM: dilated cardiomyopathy; GW: general ward; HF: heart failure; ICU: intensive care unit; IHD: ischemic heart disease; NYHA: New York Heart Association; PH: pulmonary hypertension; pre-ADL: activity of daily living at admission; PVD: peripheral vascular disease; SD: standard deviation; VHD: valvular heart disease.

### Post-admission treatments

Table 2 shows the post-admission treatments in the pre-match and matched samples. In the pre-match cohort, the use of inotrope, diuretic, aldosterone antagonist, ACE-I, beta-blocker, antiplatelet, digitalis, vasodilator, calcium-channel blocker, and statin was significantly higher, and the use of anticoagulant was significantly lower in the ICU group than in the GW group. In the matched cohort, the use of inotrope, diuretic, aldosterone antagonist, ACE-I, beta-blocker, antiplatelet, digitalis, vasodilator, calcium-channel blocker, and statin was significantly higher in the ICU group than in the GW group. In the pre-match samples, hemodiafiltration, mechanical ventilation, cardiopulmonary resuscitation, cardioversion, IABP, percutaneous coronary intervention, coronary artery bypass grafting, temporary pacing, and ECMO were more frequently performed in the ICU group, whereas after matching, hemodiafiltration, mechanical ventilation, IABP, temporary pacing, and ECMO were performed more frequently in the ICU group.

**Table 2:** Post-admission treatments in the pre-match and matched samples.

Variable	Before propensity score matching				After propensity score matching			
	All patients (n = 12231)	GW (n = 8708)	ICU (n = 3523)	P-value	All patients (n = 5970)	GW (n = 2985)	ICU (n = 2985)	P-value
**Drugs**								
**Inotrope**	2799 (22.9%)	1882 (21.6%)	917 (26.0%)	<0.001	1408 (23.6%)	654 (21.9%)	754 (25.3%)	0.002
**Diuretic**	10167 (83.1%)	7100 (81.5%)	3067 (87.0%)	<0.001	5029 (84.2%)	2426 (81.3%)	2603 (87.2%)	<0.001
**Aldosterone antagonist**	5821 (47.6%)	4045 (46.4%)	1776 (50.4%)	<0.001	2886 (48.3%)	1370 (45.9%)	1516 (50.8%)	<0.001
**ACE-I**	9132 (74.7%)	6184 (71.0%)	2948 (83.7%)	<0.001	4684 (78.5%)	2193 (73.5%)	2491 (83.5%)	<0.001
**ARB**	3766 (30.8%)	2650 (30.4%)	1116 (31.7%)	0.177	1815 (30.4%)	881 (29.5%)	934 (31.3%)	0.136
**Beta-blocker**	7792 (63.7%)	5431 (62.4%)	2361 (67.0%)	<0.001	3843 (64.4%)	1858 (62.2%)	1985 (66.5%)	<0.001
**Class III antiarrhythmic**	1094 (8.9%)	754 (8.7%)	340 (9.7%)	0.082	536 (9.0%)	255 (8.5%)	281 (9.4%)	0.239
**Antiplatelet**	4939 (40.4%)	3379 (38.8%)	1560 (44.2%)	<0.001	2541 (42.6%)	1235 (41.4%)	1306 (43.8%)	0.003
**Anticoagulant**	5711 (46.7%)	4158 (47.7%)	1553 (44.0%)	<0.001	2701 (45.2%)	1353 (45.3%)	1348 (45.2%)	0.221
**Digitalis**	1678 (13.7%)	986 (11.3%)	692 (19.6%)	<0.001	977 (16.4%)	404 (13.5%)	573 (19.2%)	<0.001
**Vasodilator**	6051 (49.5%)	3878 (44.5%)	2173 (61.6%)	<0.001	3290 (55.1%)	1469 (49.2%)	1821 (61.0%)	<0.001
**Calcium-channel blocker**	4503 (36.8%)	3027 (34.8%)	1476 (41.9%)	<0.001	2357 (39.5%)	1123 (37.6%)	1234 (41.3%)	0.003
**Statin**	3749 (30.6%)	2524 (29.0%)	1225 (34.8%)	<0.001	1951 (32.7%)	931 (31.2%)	1020 (34.2%)	0.014
**Therapies**								
**Hemodiafiltration**	462 (3.8%)	279 (3.2%)	183 (5.2%)	<0.001	253 (4.2%)	111 (3.7%)	142 (4.8%)	0.046
**Mechanical ventilation**	1707 (14.0%)	687 (7.9%)	1020 (29.0%)	<0.001	1087 (18.2%)	306 (10.3%)	781 (26.2%)	<0.001
**CPR**	36 (0.3%)	18 (0.2%)	18 (0.5%)	0.005	24 (0.4%)	12 (0.4%)	12 (0.4%)	1.000
**Cardioversion**	269 (2.2%)	164 (1.9%)	105 (3.0%)	<0.001	143 (2.4%)	60 (2.0%)	83 (2.9%)	0.052
**IABP**	73 (0.6%)	24 (0.3%)	49 (1.4%)	<0.001	54 (0.9%)	19 (0.6%)	35 (1.2%)	0.029
**PCI**	437 (3.6%)	277 (3.2%)	160 (4.5%)	<0.001	266 (4.5%)	130 (4.4%)	136 (4.6%)	0.707
**Ablation**	90 (0.7%)	57 (0.7%)	33 (0.9%)	0.098	55 (0.9%)	25 (0.8%)	30 (1.0%)	0.498
**CABG**	29 (0.2%)	14 (0.2%)	15 (0.4%)	0.006	22 (0.4%)	9 (0.3%)	13 (0.4%)	0.393
**Temporary pacing**	115 (0.9%)	56 (0.6%)	59 (1.7%)	<0.001	84 (1.4%)	32 (1.1%)	52 (1.7%)	0.028
**ECMO**	103 (0.8%)	44 (0.5%)	59 (1.7%)	<0.001	71 (1.2%)	26 (0.9%)	45 (1.5%)	0.023

Data are shown as numbers (%).

ACE-I: angiotensin-converting enzyme inhibitor; ARB: angiotensin II receptor blocker; CABG: coronary artery bypass grafting; CPR: cardiopulmonary resuscitation; ECMO: extracorporeal membrane oxygenation; IABP: intra-aortic balloon pumping; PCI: percutaneous coronary intervention.

### Comparison of outcomes

**Fig 1:**
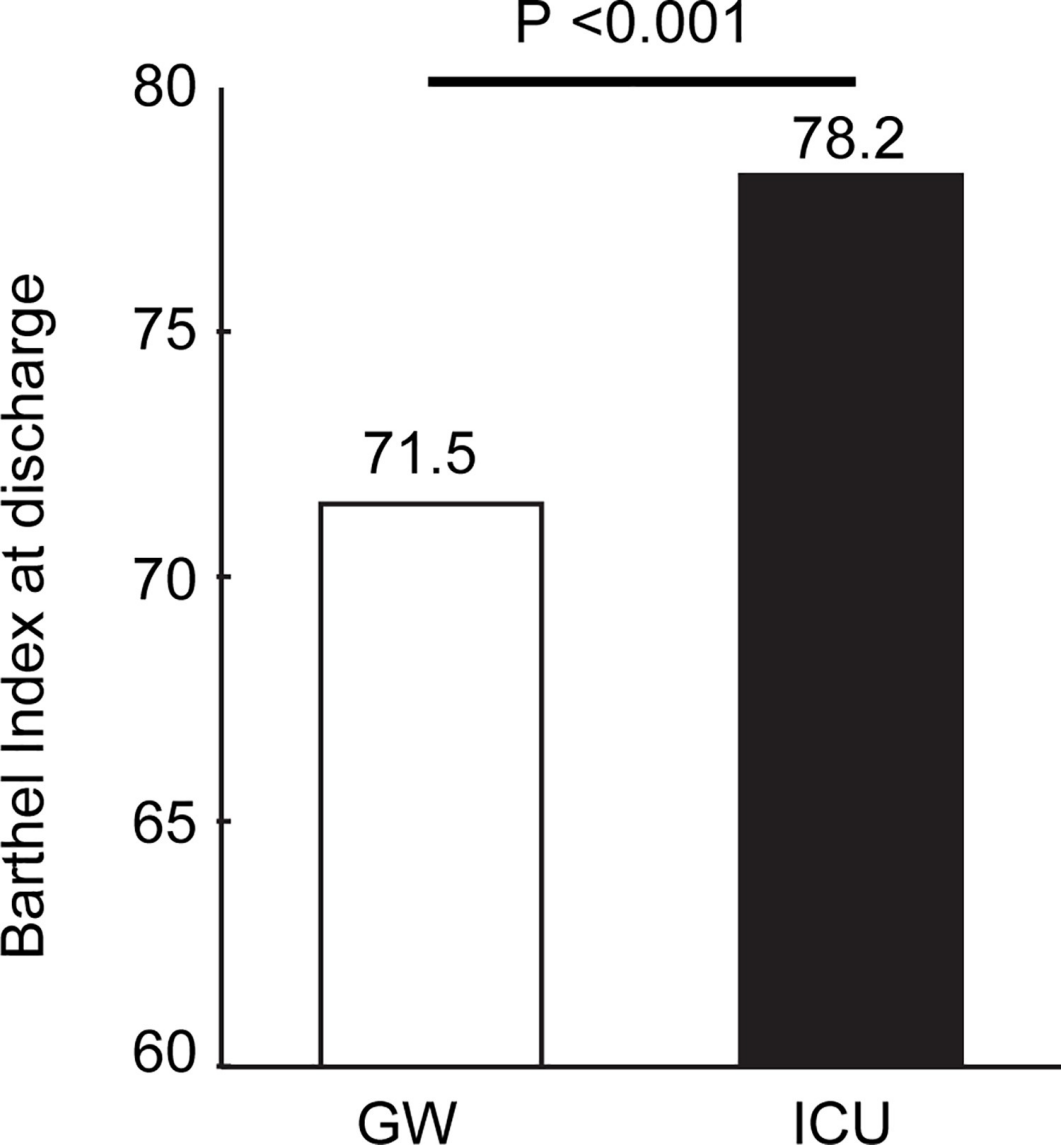
Barthel Index at discharge in the matched samples (N = 2985 patients each). GW: general ward; ICU: intensive care unit.

The propensity score analysis showed that post-ADL was significantly higher in the ICU group [mean (SD), pre-ADL: 44.5 (39.7) points; post-ADL: 78.2 (31.2) points] than in the GW group [pre-ADL: 44.7 (38.6) points; post-ADL: 71.5 (35.3) points] (Table 1 and Fig 1). The difference in mean post-ADL levels was 6.7 (95% CI: 5.1–8.4) points. Post-ADL before matching was significantly higher in the GW group than in the ICU group (pre-ADL: 80.5 (30.2) vs. 77.2 (32.0) points; P <0.001, N = 8708 vs. 3523). These findings were supported by a series of sensitivity analyses: 1) propensity score matching analyses among various NYHA categories (S1 Table); 2) propensity score matching analysis where changes in ADL from admission to discharge were used as the outcome variable (S2 Table); 3) multivariable linear regression analysis, where post-ADL was used as dependent variable and baseline confounding factors, including pre-ADL, were used as independent variables, among the entire (unmatched) patient cohort (S3 Table); 4) propensity score matching analysis in NYHA I–Ⅲ patients including those who died in the hospital (S4 Table).

For the secondary outcomes, in the pre-match samples, there was no significant difference in LOS between the groups. However, after matching, the LOS of the ICU group was significantly shorter than that of the GW group. In both the pre-match and matched cohorts, the expenses of the ICU group were significantly higher than those of the GW group (S5 Table).

### Stratified analyses

We further explored effect modifications by baseline variables on the post-ADL difference. The multivariate analysis showed significant effect modifications by age ambulance use, pre-ADL, NYHA class, diabetes, dyslipidemia, cerebrovascular disease, valvular heart disease, dilated cardiomyopathy, and dementia (S6 Table). The propensity score matching analyses in the stratified groups by these variables supported these findings (Table 3). In patients with pre-ADL ≥60 points, post-ADL was significantly lower in the ICU group. In patients with age ≤59 years and cerebrovascular diseases, there was no significant difference in post-ADL between the ICU and GW groups, whereas in other cohorts, post-ADL was significantly higher in the ICU group.

**Table 3:** Stratified analysis of ADL (Barthel Index) at discharge by age, ambulance use, pre-ADL, NYHA class at admission, diabetes, dyslipidemia, cerebrovascular disease, valvular heart disease, dilated cardiomyopathy, and dementia.

Variable	No. of patients	GW	ICU	Difference in mean (95% CI)	P-value
**All**	2985 vs. 2985	71.5 (35.0)	78.2 (31.0)	6.7 (5.1, 8.4)	<0.001
**Age (years)**					
**<40**	15 vs. 15	96.0 (9.7)	92.7 (17.1)	-3.4 (-13.9, 7.2)	0.516
**40–59**	226 vs. 226	93.8 (17.0)	93.7 (17.7)	-0.2 (-3.4, 3.1)	0.924
**60–79**	1112 vs. 1112	81.5 (29.7)	88.0 (24.2)	6.5 (4.3, 8.8)	<0.001
**≥80**	1572 vs. 1572	61.7 (36.9)	68.8 (34.4)	7.1 (4.6, 9.6)	<0.001
**Ambulance use**					
**yes**	1486 vs. 1486	68.8 (36.7)	73.2 (34.3)	4.4 (1.8, 6.9)	0.001
**no**	1451 vs. 1451	74.7 (33.3)	82.3 (28.0)	7.6 (5.3, 9.8)	<0.001
**Pre-ADL**					
**<60 points**	1877 vs. 1877	59.5 (37.4)	68.5 (34.6)	9.0 (6.7, 11.3)	<0.001
**≥60 points**	1049 vs. 1049	94.9 (12.7)	93.7 (15.9)	-1.3 (-2.5, -0.0)	0.046
**NYHA class at admission**					
**I**	327 vs. 327	70.2 (35.0)	80.2 (31.3)	10.0 (4.9, 15.1)	<0.001
**II**	1023 vs. 1023	71.9 (34.8)	78.4 (30.6)	6.6 (3.7, 9.4)	<0.001
**III**	1640 vs. 1640	71.8 (35.8)	77.5 (31.9)	5.8 (3.5, 8.1)	<0.001
**Diabetes**					
**yes**	858 vs. 858	75.7 (33.2)	83.3 (27.5)	7.7 (4.8, 10.5)	<0.001
**no**	2099 vs. 2099	69.8 (35.8)	76.0 (32.4)	6.2 (4.1, 8.3)	<0.001
**Dyslipidemia**					
**yes**	655 vs. 655	81.9 (29.3)	87.4 (24.7)	5.5 (2.6, 8.5)	<0.001
**no**	2309 vs. 2309	69.1 (35.8)	75.6 (32.4)	6.7 (4.7, 8.7)	<0.001
**Cerebrovascular disease**					
**yes**	216 vs. 216	59.4 (37.2)	62.5 (36.8)	3.1 (-4.0, 10.1)	0.392
**no**	2761 vs. 2761	73.0 (34.9)	79.6 (30.4)	6.6 (4.9, 8.3)	<0.001
**VHD**					
**yes**	433 vs. 433	71.4 (34.6)	80.9 (29.0)	9.5 (5.3, 13.8)	<0.001
**no**	2526 vs. 2526	71.9 (35.5)	77.8 (31.7)	5.9 (4.1, 7.8)	<0.001
**DCM**					
**yes**	122 vs. 122	84.0 (26.5)	93.0 (18.5)	9.0 (3.3, 14.8)	0.002
**no**	2846 vs. 2846	70.4 (35.6)	77.4 (31.7)	7.1 (5.3, 8.8)	<0.001
**Dementia**					
**yes**	863 vs. 863	53.0 (38.6)	60.5 (36.5)	7.5 (4.0, 11.1)	<0.001
**no**	2112 vs. 2112	79.3 (30.2)	85.1 (26.1)	5.8 (4.0, 7.5)	<0.001

Data are shown as number or mean (standard deviation). In each stratified group, we created propensity scores using the conditional probability of ICU admission on day 1, estimated by a logistic regression model with ICU admission as the dependent variable and all measured baseline variables, except for a stratified group variable, as independent variables. After propensity score matching, the post-ADLs of patients in the ICU and GW were compared.

DCM: dilated cardiomyopathy; NYHA: New York Heart Association; pre-ADL: ADL at admission; VHD: valvular heart disease.

## Discussion

This retrospective cohort study investigated the effect of direct ICU/CCU admission compared with GW admission in terms of ADL improvement in ADHF patients. Using propensity score methods, we found that improvement from pre-ADL to post-ADL was significantly higher in the ICU group.

Post-ADL of ADHF patients is closely related to short-and long-term mortality and readmission [24], and improvement in ADL levels leads to lower mortality in patients with impaired ADL at baseline [17]. No report has precisely described the minimal clinically important difference for heart failure patients while in stroke patients, it has been estimated to be 1.85 points [25]; therefore, a post-ADL difference between the ICU and GW groups of 6.7 points can be strongly considered as a clinically important difference with a notable impact on patient prognosis [14].

Several reasons may account for the favorable effects of early ICU/CCU admission. In ICU/CCU, the appropriate use of drug combinations and the start of intensive care, such as mechanical circulatory support for ADHF via close assessment of the circulatory status, is possible [6]. Even after propensity score matching, the use of multiple medications was significantly higher in the ICU group, showing that optimal medical therapy was better applied in the ICU group. In addition, most ICU-level therapies were performed more frequently in the ICU group. In particular, a large difference was seen in the use of mechanical ventilation (GW vs. ICU, 10.0% vs. 25.5%). The rapid introduction of ventilatory support and appropriate use of non-invasive and invasive ventilation are critical for rapid stabilization, preventing a worsening of events and unnecessary rest [6]. The LOS in the ICU group was significantly shorter (approximately 2 days less) than that in the GW group, and the reduction of unnecessary stay in hospitals may also partially account for the better post-ADL recovery. In addition to medical treatments, the difference in supportive care (which requires very frequent communication of complex and important information between multiple health care providers) exists between ICU/CCU and GW, which may lead to differences in non-cardiac adverse events, including falls, pressure ulcers, and fluid and electrolyte disorders [26].

The stratified group analyses showed significant effect modifications by several confounding factors. Previous reports described that some of the outcomes in heart failure patients differed with age and sex [27, 28]. However, in this study, sex had no interaction with ICU/CCU admission (S6 Table). Our results are consistent with the study by Rossello et al., who showed that the outcomes of ADHF patients stratified by sex might not be significantly different [29]. In contrast, patients aged ≥60 years demonstrated more beneficial effects than patients aged <60 years.

For patients with cerebrovascular disease, ADL level might be impaired before ADHF occurs, and cerebrovascular disease could persist even after ADHF recovery.

When patients were categorized based on pre-ADL, patients with normal and mild impairments had harmful effects from ICU admission, whereas patients with severe impairment demonstrated a clear benefit. The BI score at ED visit is a strong independent predictor of all-cause 30-day mortality in patients with acute heart failure admitted to ED [24, 30], and our results showed that pre-ADL level was also a strong predictor of post-ADL level. For young and physically fit patients, ICU/CCU admission may lead to unnecessary rest or restriction and post-intensive care syndrome [31], counteracting the beneficial effect of rapid ICU-level intervention. These results suggest that pre-ADL level can be a useful tool for the triage of ADHF patients to ICU/CCU admission.

For NYHA class, the patients in NYHA class I-III gained substantial benefit from ICU admission, but NYHA Ⅰ or Ⅱ cohort may benefit more from early stoppage of ADHF deteriorative effect on ADL through ICU admission. For other comorbidities, the difference between each stratified group may come from the comorbidities themselves or the difference in each cohort background, including age and pre-ADL [32]. For the ambulance category, ADHF patients brought in by ambulance demonstrated a lower impact of direct ICU admission. The reason for this is unclear, but early vital support in the ambulance may partially offset the positive effects of early ICU admission.

In this study, we excluded ADHF patients with very severe decompensation (NYHA IV patients) because such patients may not have been well matched between the ICU and GW groups. However, beneficial effects were also seen with different exclusion criteria, including the NYHA IV only cohort. These results further support the beneficial effect of ICU admission in patients with intermediate-risk ADHF to relatively more severe cohorts. BI can be only measured in survivors; therefore, if there are more deaths in one group than in the other, the estimation might be biased [33]. Considering the possibility of this competing risk of death, we performed a sensitivity analysis that included patients who died in hospital, and the trend was the same as the analyses that excluded dead patients.

The beneficial effect of ICU admission should be reviewed based on cost-effectiveness. The expenses of the ICU group were significantly higher than those of the GW group (353408 yen, approximately $3213, assuming $1 equals 110 yen), for a post-ADL improvement of 71.5–78.2 points. If the cohort was aged ≥ 60 years, with pre-ADL <60, and without cerebrovascular disease, the extra cost for the ICU group was 358352 yen (approximately $3258) for a post-ADL improvement of 59.8–68.6 points (S7 Table). Many studies regarding ICU-level therapy costs reported favorable cost-effectiveness of <$50,000 per life-year or quality-adjusted life-year. Although quality of life and ADL are related [13], the direct translation of these ADL improvements to quality-adjusted life-year was impractical. However, post-ADL <60 points has been reported to be associated with a significantly higher risk of mortality and readmission compared to post-ADL >60 points [17, 24]. Therefore, the transition from the high-risk category (post-ADL <60 points) to the low-risk category (post-ADL ≥60 points) by ICU admission could partially justify the cost increase.

The present study has several limitations. First, since the study used administrative DPC data, it lacked detailed information, such as laboratory data and radiographic and echocardiographic evaluations. The direct comparison of basic characteristics of heart failure patients to those reported in former studies using several criteria for patient prognosis was unavailable. Second, this study was an observational study and was not randomized. Although we used adjusting methods, unmeasured confounding factors, such as vital signs and examination data, may have affected the relationship between ICU admission and post-ADL. Third, the information on outcome measurements after discharge was lacking [33]. Better ADL status may lead to the prevention of readmission and mortality; the effect of early ICU entry on longer-term prognosis was not evaluated. Fourth, the percentage of NYHA-unknown patients was relatively high. However, in each NYHA category (NYHA I, II, III, IV, NYHA I–II, NYHA I–III, NYHA I–IV, and NYHA I–IV and unknown), the same results were observed, suggesting that the beneficial effect of ICU admission can be generalized. Fifth, in this study, we did not consider ICU admission after the second day of admission. The rate of GW to ICU transition and readmission after ICU-GW transition could have affected the primary and secondary outcomes. Sixth, the usage of pulmonary artery catheter, which has been reported to be effective in the management of severe ADHF patients [34] and can be performed only in the ICU but not in the GW in many settings, was not included in our analysis because of data unavailability. Finally, the relatively long hospital stay of our patients compared to that in the Western countries may affect the post-ADL, and caution must be taken when applying our findings to different cohorts of patients [32].

## Conclusions

This retrospective cohort study found that early ICU/CCU entry was beneficially associated with post-ADL in patients with emergency ADHF admission.

## Supporting information

S1 Fig Flowchart for patient selection in the study. ADL: activities of daily living; BMI: body mass index; GW: general ward; ICU: intensive care unit; NYHA: New York Heart Association.
(TIF)

S1 Table ADL at discharge in the pre-match and matched samples of different NYHA cohorts. Data are shown as numbers and mean (standard deviation). * NYHA I-IV patients excluding those who received respirator treatment on admission day 1. ADL: activities of daily living; GW: general ward; ICU: intensive care unit; NYHA: New York Heart Association.
(DOCX)

S2 Table Changes in ADL from admission to discharge. Data are shown as mean (standard deviation). ADL: activities of daily living; post-ADL: ADL at discharge; pre-ADL: ADL at admission; ΔADL: post-ADL − pre-ADL; GW: general ward; ICU: intensive care unit; NYHA: New York Heart Association.
(DOCX)

S3 Table Multivariable analysis for factors associated with ADL at discharge. BMI: body mass index; CI, confidence interval; COPD: chronic obstructive pulmonary disease; CRD: chronic renal disease; DCM: dilated cardiomyopathy; HF: heart failure; ICU: intensive care unit; IHD: ischemic heart disease; NYHA: New York Heart Association; PH: pulmonary hypertension; pre-ADL: activity of daily living at admission; PVD: peripheral vascular disease; VHD: valvular heart disease.
(DOCX)

S4 Table ADL at discharge in the pre-match and matched samples of cohorts including in-hospital death cases. Data are shown as mean (standard deviation). In the prematched samples, the numbers of ADHF patients who died in hospital were 55 (0.6%) in GW and 10 (0.3%) in ICU. In the matched samples, the numbers were 8 (0.3%) in GW and 9 (0.3%) in ICU. ADL: activities of daily living; post-ADL: ADL at discharge; GW: General ward; ICU: Intensive care unit.
(DOCX)

S5 Table Length of stay (LOS) and admission expense (expense) in the pre-match and matched samples. Data are shown as mean (standard deviation). GW, general ward; ICU, intensive care unit.
(DOCX)

S6 Table Multivariable analysis for interaction with ICU admission. BMI: body mass index; COPD: chronic obstructive pulmonary disease; CRD: chronic renal disease; DCM: dilated cardiomyopathy; HF: heart failure; ICU: intensive care unit; IHD: ischemic heart disease; NYHA: New York Heart Association; PH: pulmonary hypertension; pre-ADL: activity of daily living at admission; PVD: peripheral vascular disease; VHD: valvular heart disease.
(DOCX)

S7 Table Activities of daily living at discharge (post-ADL), length of stay (LOS) and admission expense (expense) in the matched cohort of patients with ADL < 60, age ≥ 60 years, and without cerebrovascular diseases. Data are shown as mean (standard deviation).
(DOCX)
