# Supplementary figures and images for: Association between early intensive care or coronary care unit admission and post-discharge performance of activities of daily living in patients with acute decompensated heart failure
Source: PLoS One. 2021 May 10;16(5):e0251505. doi: 10.1371/journal.pone.0251505 (PMC8109822; doi:10.1371/journal.pone.0251505)

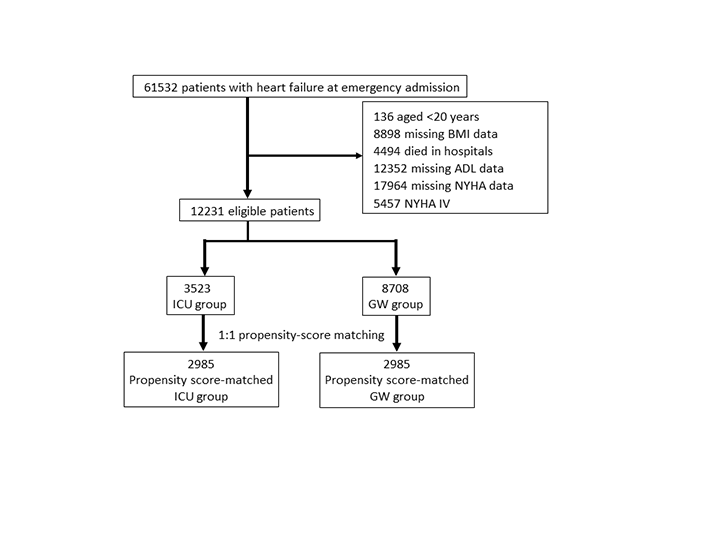

Supplement: S1 Fig — ADL: activities of daily living; BMI: body mass index; GW: general ward; ICU: intensive care unit; NYHA: New York Heart Association. (TIF) [file pone.0251505.s001.TIF]
