# Supplementary material for: Association between early intensive care or coronary care unit admission and post-discharge performance of activities of daily living in patients with acute decompensated heart failure
Source: PLoS One. 2021 May 10;16(5):e0251505. doi: 10.1371/journal.pone.0251505 (PMC8109822; doi:10.1371/journal.pone.0251505)
Supplement: S1 Table — Data are shown as numbers and mean (standard deviation). * NYHA I-IV patients excluding those who received respirator treatment on admission day 1. ADL: activities of daily living; GW: general ward; ICU: intensive care unit; NYHA: New York Heart Association. (DOCX) [file pone.0251505.s002.docx]

**S1 Table**

| **Variable** | **Before propensity score matching** | | | | **After propensity score matching** | | | |
| --- | --- | --- | --- | --- | --- | --- | --- | --- |
|  | **No. of patients** | **GW** | **ICU** | **P-value** | **No. of patients** | **GW** | **ICU** | **P-value** |
| **NYHA I-III (main analysis)** | 8708 vs. 3523 | 80.5 (30.2) | 77.2 (32.0) | <0.001 | 2985 vs. 2985 | 71.5 (35.0) | 78.2 (31.0) | <0.001 |
| **NYHA I-IV + unknown** | 23289 vs. 12068 | 78.4 (32.3) | 76.7 (32.7) | <0.001 | 9427 vs. 9427 | 70.2 (36.6) | 77.6 (32.0) | <0.001 |
| **NYHA I-IV** | 11562 vs. 5962 | 78.9 (31.7) | 76.7 (32.6) | <0.001 | 4814 vs. 4814 | 70.9 (36.1) | 77.5 (31.8) | <0.001 |
| **NYHA I­-IV without respirator *** | 10894 vs. 4206 | 78.8 (31.7) | 75.5 (33.1) | <0.001 | 3892 vs. 3892 | 70.1 (36.3) | 76.0 (32.6) | <0.001 |
| **NYHA I-II** | 4363 vs. 1582 | 81.3 (29.4) | 78.0 (31.1) | <0.001 | 1331 vs. 1331 | 71.3 (34.5) | 79.3 (30.3) | <0.001 |
| **NYHA IV** | 2939 vs. 2468 | 73.7 (35.3) | 76.1 (33.4) | 0.013 | 1803 vs. 1803 | 70.7 (37.3) | 75.7 (33.4) | <0.001 |
