# Supplementary material for: Association between early intensive care or coronary care unit admission and post-discharge performance of activities of daily living in patients with acute decompensated heart failure
Source: PLoS One. 2021 May 10;16(5):e0251505. doi: 10.1371/journal.pone.0251505 (PMC8109822; doi:10.1371/journal.pone.0251505)
Supplement: S2 Table — Data are shown as mean (standard deviation). ADL: activities of daily living; post-ADL: ADL at discharge; pre-ADL: ADL at admission; ΔADL: post-ADL − pre-ADL; GW: general ward; ICU: intensive care unit; NYHA: New York Heart Association. (DOCX) [file pone.0251505.s003.docx]

**S2 Table**

| **Variable** | **Before propensity score matching** | | | **After propensity score matching** | | |
| --- | --- | --- | --- | --- | --- | --- |
|  | **GW**  **(n = 8708)** | **ICU**  **(n = 3523)** | **P-value** | **GW**  **(n = 2984)** | **ICU**  **(n = 2984)** | **P-value** |
| **ΔADL** | 14.9 (28.6) | 38.2 (40.1) | <0.001 | 26.7 (35.0) | 33.7 (39.0) | <0.001 |
