# Supplementary material for: Association between early intensive care or coronary care unit admission and post-discharge performance of activities of daily living in patients with acute decompensated heart failure
Source: PLoS One. 2021 May 10;16(5):e0251505. doi: 10.1371/journal.pone.0251505 (PMC8109822; doi:10.1371/journal.pone.0251505)
Supplement: S3 Table — BMI: body mass index; CI, confidence interval; COPD: chronic obstructive pulmonary disease; CRD: chronic renal disease; DCM: dilated cardiomyopathy; HF: heart failure; ICU: intensive care unit; IHD: ischemic heart disease; NYHA: New York Heart Association; PH: pulmonary hypertension; pre-ADL: activity of daily living at admission; PVD: peripheral vascular disease; VHD: valvular heart disease. (DOCX) [file pone.0251505.s004.docx]

**S3 Table**

|  | **Multivariable analysis** | |
| --- | --- | --- |
| **Variables** | **β (95%CI)** | **P-value** |
| **Use of ICU on admission** | 0.09 (0.08, 0.11) | <0.001 |
| **Age** | -0.16 (-0.18, -0.15) | <0.001 |
| **Male sex** | 0.04 (0.03, 0.06) | <0.001 |
| **BMI** | 0.05 (0.03, 0.06) | <0.001 |
| **Ambulance use** | 0.00 (-0.01, 0.02) | 0.865 |
| **Weekend admission** | 0.01 (0.00, 0.03) | 0.032 |
| **History of HF admission** | 0.00 (-0.01, 0.02) | 0.703 |
| **pre-ADL, mean** | 0.44 (0.42, 0.46) | <0.001 |
| **NYHA class at admission** |  |  |
| **I** | ref |  |
| **II** | 0.00 (-0.02, 0.02) | 0.874 |
| **III** | 0.00 (-0.02, 0.02) | 0.823 |
| **Impairment in consciousness** | -0.11(-0.13, -0.10) | <0.001 |
| **Hypertension** | 0.03 (0.02, 0.05) | <0.001 |
| **Diabetes** | 0.01 (-0.01, 0.02) | 0.269 |
| **Dyslipidemia** | 0.05 (0.03, 0.06) | <0.001 |
| **Cerebrovascular disease** | -0.05 (-0.06, -0.03) | <0.001 |
| **Atrial fibrillation** | -0.02 (-0.05, 0.01) | 0.284 |
| **Cardiac arrhythmia** | 0.06 (0.03, 0.09) | <0.001 |
| **IHD** | 0.05 (0.04, 0.06) | <0.001 |
| **VHD** | 0.03 (0.02, 0.04) | <0.001 |
| **DCM** | 0.02 (0.01, 0.04) | <0.001 |
| **PVD** | 0.00 (-0.01, 0.01) | 0.992 |
| **PH** | -0.01 (-0.02, 0.01) | 0.401 |
| **Congenital heart disease** | 0.00 (-0.01, 0.01) | 0.961 |
| **Pneumonia** | -0.03 (-0.05, -0.02) | <0.001 |
| **COPD or asthma** | 0.02 (0.01, 0.03) | 0.003 |
| **CRD** | 0.00 (-0.01, 0.02) | 0.559 |
| **Anemia** | 0.00 (-0.02, 0.01) | 0.780 |
| **Cancer** | -0.02 (-0.03, 0.00) | 0.008 |
| **Disuse** | -0.02 (-0.03, -0.01) | 0.002 |
| **Dementia** | -0.19 (-0.20, -0.17) | <0.001 |
| **Annual hospital volume, case/year** |  |  |
| **Quartile 1 (<59)** | ref |  |
| **Quartile 2 (59–126)** | 0.02 (-0.01, -0.04) | 0.187 |
| **Quartile 3 (127–210)** | -0.01 (-0.04, 0.01) | 0.262 |
| **Quartile 4 (≥211)** | 0.00 (-0.00, 0.00) | 0.801 |
