# Supplementary material for: Association between early intensive care or coronary care unit admission and post-discharge performance of activities of daily living in patients with acute decompensated heart failure
Source: PLoS One. 2021 May 10;16(5):e0251505. doi: 10.1371/journal.pone.0251505 (PMC8109822; doi:10.1371/journal.pone.0251505)
Supplement: S4 Table — Data are shown as mean (standard deviation). In the prematched samples, the numbers of ADHF patients who died in hospital were 55 (0.6%) in GW and 10 (0.3%) in ICU. In the matched samples, the numbers were 8 (0.3%) in GW and 9 (0.3%) in ICU. ADL: activities of daily living; post-ADL: ADL at discharge; GW: General ward; ICU: Intensive care unit. (DOCX) [file pone.0251505.s005.docx]

**S4 Table**

| **Variable** | **Before propensity score matching** | | | **After propensity score matching** | | |
| --- | --- | --- | --- | --- | --- | --- |
|  | **GW**  **(n = 8763)** | **ICU**  **(n = 3533)** | **P-value** | **GW**  **(n = 2999)** | **ICU**  **(n = 2999)** | **P-value** |
| **Post-ADL** | 79.3 (31.5) | 76.9 (32.3) | <0.001 | 70.5 (35.6) | 77.8 (31.7) | <0.001 |
