# Supplementary material for: Association between early intensive care or coronary care unit admission and post-discharge performance of activities of daily living in patients with acute decompensated heart failure
Source: PLoS One. 2021 May 10;16(5):e0251505. doi: 10.1371/journal.pone.0251505 (PMC8109822; doi:10.1371/journal.pone.0251505)
Supplement: S5 Table — Data are shown as mean (standard deviation). GW, general ward; ICU, intensive care unit. (DOCX) [file pone.0251505.s006.docx]

**S5 Table**

| Variable | **Before propensity score matching** | | | **After propensity score matching** | | |
| --- | --- | --- | --- | --- | --- | --- |
|  | **GW**  **(n = 8708)** | **ICU**  **(n = 3523)** | **P-value** | **GW**  **(n = 2984)** | **ICU**  **(n = 2984)** | **P-value** |
| LOS (days) | 20.1 (18.2) | 19.7 (16.6) | 0.201 | 21.4 (19.0) | 19.4 (15.5) | <0.001 |
| Expense (yen) | 935982.5 (1038337.7) | 1394822.7 (1174540.4) | <0.001 | 1023418.1 (1075369.8) | 1376825.9 (1144371.2) | <0.001 |
