# Supplementary material for: Association between early intensive care or coronary care unit admission and post-discharge performance of activities of daily living in patients with acute decompensated heart failure
Source: PLoS One. 2021 May 10;16(5):e0251505. doi: 10.1371/journal.pone.0251505 (PMC8109822; doi:10.1371/journal.pone.0251505)
Supplement: S6 Table — BMI: body mass index; COPD: chronic obstructive pulmonary disease; CRD: chronic renal disease; DCM: dilated cardiomyopathy; HF: heart failure; ICU: intensive care unit; IHD: ischemic heart disease; NYHA: New York Heart Association; PH: pulmonary hypertension; pre-ADL: activity of daily living at admission; PVD: peripheral vascular disease; VHD: valvular heart disease. (DOCX) [file pone.0251505.s007.docx]

**S6 Table**

|  | **Multivariable analysis** |
| --- | --- |
| **Variables** | **P-value for interaction** |
| **Age** | 0.004 |
| **Male sex** | 0.661 |
| **BMI** | 0.267 |
| **Ambulance use** | 0.002 |
| **Weekend admission** | 0.162 |
| **History of HF admission** | 0.240 |
| **pre-ADL, mean** | <0.001 |
| **NYHA class at admission** |  |
| **I** | ref |
| **II** | 0.233 |
| **III** | 0.041 |
| **Impairment in consciousness** | 0.258 |
| **Hypertension** | 0.074 |
| **Diabetes** | 0.007 |
| **Dyslipidemia** | 0.009 |
| **Cerebrovascular disease** | 0.008 |
| **Atrial fibrillation** | 0.864 |
| **Cardiac arrhythmia** | 0.821 |
| **IHD** | 0.367 |
| **VHD** | 0.005 |
| **DCM** | 0.009 |
| **PVD** | 0.895 |
| **PH** | 0.752 |
| **Congenital heart disease** | 0.529 |
| **Pneumonia** | 0.072 |
| **COPD or asthma** | 0.782 |
| **CRD** | 0.567 |
| **Anemia** | 0.798 |
| **Cancer** | 0.980 |
| **Disuse** | 0.509 |
| **Dementia** | <0.001 |
| **Annual hospital volume, case/year** |  |
| **Quartile 1 (<59)** | ref |
| **Quartile 2 (59–126)** | 0.442 |
| **Quartile 3 (127–210)** | 0.373 |
| **Quartile 4 (≥211)** | 0.536 |
