# Supplementary material for: Association between early intensive care or coronary care unit admission and post-discharge performance of activities of daily living in patients with acute decompensated heart failure
Source: PLoS One. 2021 May 10;16(5):e0251505. doi: 10.1371/journal.pone.0251505 (PMC8109822; doi:10.1371/journal.pone.0251505)
Supplement: S7 Table — Data are shown as mean (standard deviation). (DOCX) [file pone.0251505.s008.docx]

**S7 Table**

| Variable | **After propensity score matching** | | |
| --- | --- | --- | --- |
|  | **GW**  **(n = 1612)** | **ICU**  **(n = 1612)** | **P-value** |
| post-ADL | 59.8 (37.2) | 68.6 (34.2) | <0.001 |
| LOS (days) | 23.7 (20.2) | 20.9 (17.2) | <0.001 |
| Expense (yen) | 1041530.3 (1075369.8) | 1399882.0 (1086061.4) | <0.001 |
